# Influence of Mycotoxins and a Mycotoxin Adsorbing Agent on the Oral Bioavailability of Commonly Used Antibiotics in Pigs

**DOI:** 10.3390/toxins4040281

**Published:** 2012-04-24

**Authors:** Joline Goossens, Virginie Vandenbroucke, Frank Pasmans, Siegrid De Baere, Mathias Devreese, Ann Osselaere, Elin Verbrugghe, Freddy Haesebrouck, Sarah De Saeger, Mia Eeckhout, Kris Audenaert, Geert Haesaert, Patrick De Backer, Siska Croubels

**Affiliations:** 1 Department of Pharmacology, Toxicology and Biochemistry, Faculty of Veterinary Medicine, Ghent University, Salisburylaan 133, 9820 Merelbeke, Belgium; Email: Virginie.Vandenbroucke@ugent.be (V.V.); Siegrid.DeBaere@ugent.be (S.D.B.); Mathias.Devreese@ugent.be (M.D.); Ann.Osselaere@ugent.be (A.O.); Patrick.DeBacker@ugent.be (P.D.B.); Siska.Croubels@ugent.be (S.C.); 2 Department of Pathology, Bacteriology and Avian Diseases, Faculty of Veterinary Medicine, Ghent University, Salisburylaan 133, 9820 Merelbeke, Belgium; Email: Frank.Pasmans@ugent.be (F.P.); Elin.Verbrugghe@ugent.be (E.V.); Freddy.Haesebrouck@ugent.be (F.H.); 3 Department of Bioanalysis, Faculty of Pharmaceutical Sciences, Ghent University, Harelbekestraat 72, 9000 Gent, Belgium; Email: Sarah.DeSaeger@ugent.be (S.D.S.); 4 Department of Food Science and Technology, Faculty of Applied Bioscience Engineering, University College Ghent, Valentin Vaerwyckweg 1, 9000 Gent, Belgium; Email: Mia.Eeckhout@hogent.be (M.E.); 5 Department of Plant Production, Faculty of Applied Bioscience Engineering, University College Ghent, Valentin Vaerwyckweg 1, 9000 Gent, Belgium; Email: Kris.Audenaert@hogent.be (K.A.); Geert.Haesaert@hogent.be (G.H.)

**Keywords:** mycotoxins, mycotoxin binder, antibiotics, pigs, interaction, safety testing

## Abstract

It is recognized that mycotoxins can cause a variety of adverse health effects in animals, including altered gastrointestinal barrier function. It is the aim of the present study to determine whether mycotoxin-contaminated diets can alter the oral bioavailability of the antibiotics doxycycline and paromomycin in pigs, and whether a mycotoxin adsorbing agent included into diets interacts with those antibiotics. Experiments were conducted with pigs utilizing diets that contained blank feed, mycotoxin-contaminated feed (T-2 toxin or deoxynivalenol), mycotoxin-contaminated feed supplemented with a glucomannan mycotoxin binder, or blank feed supplemented with mycotoxin binder. Diets with T-2 toxin and binder or deoxynivalenol and binder induced increased plasma concentrations of doxycycline administered as single bolus in pigs compared to diets containing blank feed. These results suggest that complex interactions may occur between mycotoxins, mycotoxin binders, and antibiotics which could alter antibiotic bioavailability. This could have consequences for animal toxicity, withdrawal time for oral antibiotics, or public health.

## 1. Introduction

Toxigenic fungi may often colonize fodder crops and feed components. Under varied environmental conditions they can produce toxic secondary metabolites, called mycotoxins. A recent study investigated the occurrence of mycotoxins in European feed samples and concluded that 82% of the samples were contaminated with mycotoxins [1], indicating that mycotoxins are omnipresent. All farm animals can experience a negative impact from a dietary intake of mycotoxins but pigs are one of the species which are highly sensitive. The economic impact of mycotoxins includes increased mortality, increased veterinary care costs, reduced livestock production, disposal of contaminated foods and feeds and investment in research and applications to reduce the mycotoxin problem [2].

Since the gastrointestinal epithelium is the first barrier exposed to mycotoxins after ingestion of contaminated feed, research has focused on the effects on barrier integrity. *In vitro* research revealed that mycotoxins are able to increase the permeability of intestinal epithelial cell monolayers. Indeed, deoxynivalenol (DON), ochratoxin A (OTA) and patulin (PAT) compromise the intestinal barrier function by altering the tight junction complex [3,4,5,6,7,8]. This reduced expression of tight junction proteins leads to an increased passage of tracers such as fluorescein isothiocyanate (FITC)-dextran and bacteria such as *Escherichia coli* [9]. In addition, *in vivo* experiments provide compelling evidence that mycotoxins can alter intestinal functions and lead to malabsorption of nutrients like glucose [10,11].

To minimize exposure to mycotoxins, a variety of physical, chemical and biological methods has been developed in order to eliminate fungi and their mycotoxins from foods and feeds [12,13]. One of the most prominent post-harvest strategies is the use of mycotoxin adsorbents as feed additives. By including various mycotoxin adsorbing agents in the compound feed, the bioavailability of mycotoxins can decrease by reducing their uptake [14]. 

The extensive use of adsorbents in the livestock industry has led to the introduction of a wide range of new products, most of them offering high *in vitro* mycotoxin adsorption capacity. Regarding the use of these products as feed additives, a primary concern is that the *in vivo* efficacy in sequestering mycotoxins and the safety towards livestock of most of these commercial products have not yet been thoroughly tested [15]. 

The European Commission [16] recently defined a new functional group of feed additives as “substances for reduction of the contamination of feed by mycotoxins: substances that can suppress or reduce the absorption, promote the excretion or modify their mode of action” and requested technical advice from the European Food Safety Authority (EFSA) on the guidelines to be followed for authorization of these additives. EFSA stated that not only *in vitro* but also *in vivo* studies are required for the assessment of mycotoxin binders with regard to efficacy testing. A major risk for mycotoxin binders, however, is the lack of selectivity with possible consequences for nutritional aspects through interactions with dietary compounds. EFSA also reported that one of the parameters to be studied is the possible interactions of mycotoxin binders with veterinary medicinal products [17]. Additives that exert their activity mainly by binding, may affect the oral bioavailability of drugs. To our knowledge, only a few studies deal with this topic. [18] described a significantly lower maximal plasma concentration for lincomycin in broilers after pretreatment with a mycotoxin binder (*C*_max_ = 3.27 ± 0.15 µg·mL−1), compared to broilers receiving no binder in the feed (*C*_max_ = 10.65 ± 0.17 µg·mL−1). The Bureau of Veterinary Drugs of Canada [19] reported a lack of efficacy of tylosin in cattle after intake of a clay containing bentonite which binds tylosin and makes it unavailable to the animal. Shryock *et al.* [20] demonstrated that bentonite (2%), mixed in the feed, renders tilmicosin completely ineffective in broiler chickens. This resulted in the prohibition of the use of a bentonite feed additive in combination with antibiotics, growth promoters, coccidiostats and other medical substances [21]. 

Since there are no other scientific papers that investigate these possible interactions, the general aim of this study was to examine whether mycotoxins, a commercially available mycotoxin binder, or the combination of both in the feed, affect the oral bioavailability of frequently used antibiotics in pigs. It is generally accepted that, in the northern hemisphere, *Fusarium* moulds are among the most important toxigenic fungi involved in the animal feed chain [22]. Therefore the mycotoxins studied were T-2 toxin (T-2) and deoxynivalenol (DON). T-2 is the most potent and cytotoxic trichothecene [23]. Moreover it is an emerging *Fusarium* mycotoxin, for which there are no recommended maximum levels in animal feed available yet [24]. DON on the other hand, is one of the most frequent contaminants of maize and small grain cereals [25]. Both mycotoxins have already been proven in our laboratory to influence the passage of the antibiotics doxycycline and paromomycin across an intestinal epithelial monolayer of porcine origin (unpublished results). These antibiotics were also used in this *in vivo* experiment as it is a common practice in current pig husbandry to administer these veterinary drugs by ‘mass’ medication in feed and/or drinking water. 

## 2. Materials and Methods

### 2.1. Animals

Twenty-four clinically healthy 9-week-old pigs (Piétrain × Landrace, local commercial pig farm), with a mean (± SD) body weight of 22.6 ± 1.1 kg, were used in the T-2 experiment (experiment 1). The animals were randomized into a control group (*n* = 6) and three experimental groups (each *n* = 6). 

A second experiment with DON was performed with another twenty-four pigs (Piétrain × Landrace), with a mean (±SD) body weight of 23.1 ± 1.4 kg. The animals were also randomized into a control group (*n* = 6) and three experimental groups (each *n* = 6).

All the animals were weighed daily and fed, once a day, 1.5 kg of the assigned feed during the first week and 2 kg of the assigned feed during the last two weeks of the experiment. Water was available *ad libitum*. The animals were housed in groups under natural light conditions.

All animal experiments have been approved by the ethics committee of the Faculty of Veterinary Medicine, Ghent University (EC 2009/094 + expansion EC 2010/012 and expansion EC 2010/038 + EC2010/120).

### 2.2. Feed

#### 2.2.1. Experiment 1: Influence of T-2 and Mycotoxin Binder on the Oral Absorption of Doxycycline and Paromomycin

Conventional pig feed was purchased (ILVO, Melle, Belgium) and analysed for the presence of mycotoxins. Analysis with liquid chromatography tandem mass spectrometry (LC-MS/MS) [1] revealed that the feed contained 479 ± 140 µg·kg^−1^ DON and 44 ± 13 µg·kg^−1^ zearalenone. All other mycotoxins (*n* = 21) tested were below the limit of detection (LOD). Since the feed did not contain T-2 toxin, it was accepted for use in experiment 1. This feed is further referred to as blank feed and was used to feed the animals during the acclimatization period as well as to prepare the T-2 contaminated feed needed for the experiment.

To produce feed contaminated with 100 µg T-2 kg^−1^, a stock solution of 250 µg·mL^−1^ T-2 toxin was prepared by dissolving 50 mg T-2 (Sigma-Aldrich, Bornem, Belgium) in 200.0 mL ethanol (Merck, Darmstadt, Germany). The contaminated feed was produced by adding 120 mL of the stock solution to 500 g of blank feed. This premix was then mixed with 5 kg of blank feed to assure a homogeneous distribution of the toxin. The final premix was then mixed for 20 min in the total amount of feed (300 kg) needed for the experiment. To test T-2 toxin homogeneity in the feed, a sample was taken at three different locations in the batch and analysed with LC-MS/MS to determine the concentration of T-2 toxin. A mean concentration of 99 ± 13 µg·kg^−1^ T-2 was found in this T-2 contaminated feed. 

The binder used in the experiment was a commercially available glucomannan mycotoxin binder which claimed to bind T-2 and DON, added at a concentration of 2 kg per metric ton. To produce the binder supplemented feed (150 kg), binder was added to both blank feed and feed contaminated with 100 µg T-2 kg^−1^ feed and mixed for 20 min. A mean concentration of 111 ± 4 µg·kg^−1^ T-2 was found in this T-2 contaminated feed supplemented with binder.

#### 2.2.2. Experiment 2: Influence of DON and Mycotoxin Binder on the Oral Absorption of Doxycycline and Paromomycin

Conventional feed was purchased (DANIS, Koolskamp, Belgium) and analysed with LC-MS/MS for the presence of mycotoxins. As the feed only contained 11 ± 4 µg·kg^−1^ T-2 and no other mycotoxins such as DON, the feed was used to feed the animals during the acclimatization period as well as to prepare the DON contaminated feed needed for the experiment. The reference strain *Fusarium graminearum* MUCL 42841 (Mycothèque de l’Université catholique de Louvain) was used to produce DON, for inclusion in feed at a theoretical concentration of 1 mg·kg^−1^. The strain was grown in liquid mineral medium supplemented with L-arginin as a selective nitrogen source [26]. After 14 days of cultivation, the culture was filtered and centrifuged. The supernatant was freeze-dried and mixed into the blank feed (300 kg) until a final DON concentration of 1 mg·kg^−1^ was obtained. To test DON homogeneity in the feed, a sample was taken at three different locations in the batch and analysed with LC-MS/MS to determine the concentration of DON. A mean concentration of 802 ± 23 µg·kg^−1^ DON was found and the feed was accepted to be used in the experiment. Blank feed supplemented with binder (150 kg) was produced as described in experiment 1. A mean concentration of 813 ± 24 µg·kg^−1^ DON was found in this DON contaminated feed supplemented with binder.

### 2.3. Study Design

#### 2.3.1. Experiment 1

After an acclimatization period of one week, during which all animals received blank feed, the four groups of six animals received blank feed (control), feed contaminated with 99 ± 13 µg T-2 kg^−1^ feed, feed contaminated with 111 ± 4 µg T-2 kg^−1^ feed and supplemented with the mycotoxin binder or blank feed supplemented with the mycotoxin binder, respectively. 

After the intake of the experimental diet for seven days, the animals received a single oral intragastric bolus of doxycycline (Soludox 50%^®^, Eurovet, Bladel, The Netherlands) and paromomycin (Gabbrovet 70^®^, CEVA Santé Animale, Brussels, Belgium). The oral solution was prepared by dissolving the powders in tap water. Doxycycline was given at a dose of 10 mg·kg^−1^ body weight and paromomycin at 100 mg·kg^−1^ body weight. Blood samples were collected in heparinised tubes (Venoject^®^, Terumo Corp., Tokyo, Japan) by puncturing the external jugular vein with a 20 G needle (20 G, 0.9 × 38 mm, Terumo Corp., Tokyo, Japan) before and at 0.5, 1, 2, 3, 4, 6, 8, 12 and 24 h after administration. The samples were centrifuged at 2,851 *g* at 4 °C for 10 min and plasma was stored at −15 °C until assayed for paromomycin and doxycycline. 

#### 2.3.2. Experiment 2

To evaluate whether another trichothecene mycotoxin results in findings comparable to the first experiment, a second experiment was performed using the mycotoxin DON. After an acclimatization period of one week, four groups of six animals received for 13 days respectively blank feed (control), feed contaminated with 802 ± 23 µg·kg^−1^ DON, feed contaminated with 813 ± 24 µg·kg^−1^ DON and supplemented with the mycotoxin binder and blank feed supplemented with the mycotoxin binder. Subsequently the animals received a single oral bolus of doxycycline (10 mg·kg^−1^ BW) and paromomycin (100 mg·kg^−1^ BW), blood was drawn and plasma samples were collected as described in experiment 1.

### 2.4. Doxycycline Determination in Plasma

Plasma doxycycline concentration was determined by high-performance liquid chromatography (HPLC), using ultraviolet detection, based on the procedure described by Baert *et al.* [27]. 

The method was in-house validated by a set of parameters that were in compliance with the recommendations as defined in several EU documents [28,29,30,31]. The following parameters were evaluated: linearity, within-run and between-run accuracy and precision, limit of quantification (LOQ), limit of detection (LOD), specificity. Quantification was performed using matrix-matched calibration curves (concentration range: 200–1,000 ng·mL^−1^ and 2,000–10,000 ng·mL^−1^) and the correlation coefficients (*r *= 0.9963 ± 0.0030, *n* = 6 and *r* = 0.9962 ± 0.0030) and goodness-of-fit coefficients (*g* = 5.65 ± 2.49% and 5.37 ± 2.44%, *n* = 6) fell within the accepted ranges, *i.e.*, *r* ≥ 0.99 and *g *≤ 10%, respectively. The internal standard (IS, demethylchlortetracycline, chemical reference substance, European Pharmacopoeia, Strasbourg, France) was added to all samples prior to the start of the sample preparation procedure. Since the principle of internal standardization was used for quantification, no correction factor had to be applied for analyte loss during sample preparation (extraction recovery).

Within-run and between-run precision and accuracy were evaluated by analyzing 6 independently spiked samples at 2 concentration levels, *i.e.*, 1000 and 5000 ng·mL^−1^, respectively. The following mean results were obtained: within-run accuracy (*n* = 6): 908.0 ± 40.4 ng·mL^−1^ and 4955 ± 223.0 ng·mL^−1^; between-run accuracy (*n* = 6): 912.0 ± 43.3 ng·mL^−1^ and 4,810 ± 153.9 ng·mL^−1^. The results fell within the accepted ranges for accuracy (−20 to +10% of the theoretical concentration) and precision (within-run precision: relative standard deviation (RSD) ≤ RSD_max_ with RSD_max_ = 2^(1−0.5log Conc)^ × 2/3, *i.e.*, 10.7% and 8.4% at 1000 ng·mL^−1^ and 5000 ng·mL^−1^, respectively; between-run precision: RSD ≤ RSD_max_ with RSD_max_ = 2^(1−0.5log Conc)^, *i.e.*, 16.0% and 12.6% at 1,000 ng·mL^−1^ and 5,000 ng·mL^−^^1^, respectively). The LOQ was defined as the lowest concentration for which the method was validated with a within-run accuracy and precision that fell within the specified ranges. The LOQ was also the lowest point of the calibration curve and was set at 200 ng·mL^−1^ (*n* = 6, mean result: 190.2 ± 3.44 ng·mL^−1^). The LOD was defined as the concentration corresponding with a signal-to-noise ratio of 3 and was found to be 82.6 ng·mL^−1^. The specificity of the method was shown, since no peaks of endogenous interferences could be determined in blank samples.

### 2.5. Paromomycin Determination in Plasma

The concentration of paromomycin in plasma was determined by LC-MS/MS. Samples were prepared by pipetting 500 µL plasma into an Eppendorf cup (Novolab, Geraardsbergen, Belgium). Each sample was spiked with 50 µL of the IS (tobramycin, Sigma) working solution of 5 µg·mL^−1^ in HPLC grade water (VWR International, Leuven, Belgium). After vortexing briefly, 100 µL of a 20% trichloroacetic acid (TCA) solution in water were added to deproteinize. After vortexing for 15 sec, the samples were centrifuged at 7,800 *g* for 10 min. The upper layer was transferred into screw-capped polypropylene vials for HPLC and 100 µL were injected onto the LC-MS/MS system.

The HPLC system consisted of a quaternary gradient pump type P4000 and an AS3000 autosampler coupled to a LCQ^®^ mass spectrometer with an electrospray ionization source operating in the positive ionization mode (ThermoFischer Scientific, Zellik, Belgium).

Chromatographic separation was achieved using a Nucleosil column (100 mm × 3 mm i.d., dp: 5 µm, Varian) protected with a guard column of the same type (10 mm × 2 mm i.d., Varian). The mobile phase consisted of 20 mmol·L^−1^ pentafluoropropionic acid (PFPA) (Sigma-Aldrich) in water (A) and 20 mmol·L^−1^ PFPA in water/acetonitrile (50/50, v/v) (VWR International) (B). A gradient elution was performed at a flow-rate of 0.2 mL·min^−1^, *i.e.*, 0–4 min, 40% B; 4–6 min: linear gradient to 90% B, 6–6.1 min, linear gradient to 40% B, 6.1–10.5 min, 40% B. Paromomycin and the IS eluted at 5.45 and 5.68 min, respectively. A divert valve was used to direct the HPLC column effluent to the MS system from 4.5 to 7.5 min only, which prevented the MS system from quick contamination.

The method was in-house validated by the same parameters as for the DOX analysis [28,29,30,31]. Quantification was performed using matrix-matched calibration curves (concentration range: 50–5,000 ng·mL^−1^) and the correlation coefficients (*r* = 0.9981 ± 0.0005, *n* = 6) and goodness-of-fit coefficients (*g* = 8.29 ± 1.71%, *n* = 6) fell within the accepted ranges, *i.e.*, *r* ≥ 0.99 and *g* ≤ 10%, respectively. 

Within-run and between-run precision and accuracy were evaluated at 2 concentration levels, *i.e.*, 250 and 2,500 ng·mL^−1^, respectively. The following mean results were obtained: within-run accuracy and precision (n = 6): 228.1 ± 11.5 ng·mL^−1^ and 2,603 ± 191.8 ng·mL^−1^; between-run accuracy and precision (*n* = 6): 225.2 ± 15.7 ng·mL^−1^ and 2,410 ± 165.8 ng·mL^−1^. The results fell within the accepted ranges for accuracy (−20 to +10% of the theoretical concentration) and precision (RSD ≤ RSD_max_ with RSD_max_ for within-run precision: 13.1% and 9.3% and RSD_max_ for between-run precision: 19.7% and 13.9% at a concentration level of 250 ng·mL^−1^ and 2,500 ng·mL^−1^, respectively). The LOQ was set at 50 ng·mL^−1^ (*n* = 6, mean result: 46.9 ± 5.26 ng·mL^−1^), while the LOD was found to be 0.09 ng·mL^−1^. The specificity of the method was shown, since no peaks of endogenous interferences could be determined in blank samples.

### 2.6. Pharmacokinetic and Statistical Analysis

Plasma concentration *versus* time data were analyzed by means of WinNonlin^®^, Version 6.2.0 (Pharsight Corporation, Mountain View, CA, USA) software program using noncompartmental analysis. The area under the plasma concentration-time curve from dosing to the last measured concentration (AUC_0–24h_) was calculated via the trapezoidal method. Data were statistically analyzed using SPSS 17.0 software for Windows (SPSS Inc., Chicago, IL, USA). Normally distributed data were analyzed using one-way analysis of variance (ANOVA) to address the significance of difference between mean values with significance set at *P* ≤ 0.05. Bonferroni as post hoc test was used when equal variances were assessed. Not normally distributed data were analyzed using the non-parametric Kruskal-Wallis analysis, followed by a Dunn’s Multiple Comparison test.

## 3. Results

### 3.1. Experiment 1

#### 3.1.1. Intake of T-2 Contaminated Feed Supplemented with Mycotoxin Binder Results in Significant Higher Plasma Concentrations of Doxycycline

**Figure 1:**
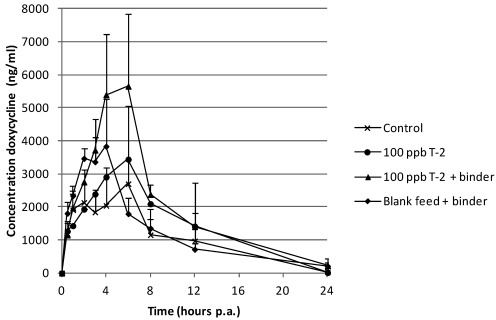
Mean plasma concentrations (+SD) in pigs after a single peroral administration of doxycycline at 10 mg kg^−1^ body weight (*n* = 6). Prior to the bolus, pigs received during 7 days, blank feed (control group), feed contaminated with 100 µg T-2 toxin per kg feed, feed contaminated with 100 µg T-2 toxin per kg feed supplemented with mycotoxin binder and blank feed supplemented with mycotoxin binder, respectively.

For the different groups, the plasma concentration-time curve of doxycycline after oral (p.o.) administration is presented in Figure 1. 

The group which received feed supplemented with 100 µg T-2 kg^−1^ feed and binder showed significantly higher plasma concentrations compared to the control group (*P* = 0.033), but not to the other experimental groups. The group which received 100 µg T-2 kg^−1^ feed and the one that received blank feed supplemented with the mycotoxin binder had plasma concentrations that were not different with the control group. The mean area under the plasma concentration-time curve (AUC_0–24h_) is summarized in Table 1. 

**Table 1:** Mean (± SD) area under the plasma concentration-time curve (AUC_0–24h_) for doxycycline in the different groups. Prior to the bolus, pigs received during 7 days, blank feed (control group), feed contaminated with 100 µg T-2 toxin per kg feed, feed contaminated with 100 µg T-2 toxin per kg feed supplemented with mycotoxin binder and blank feed supplemented with mycotoxin binder, respectively. Superscript (*) refers to a significant difference compared to the control group (*P* < 0.05).

Group	AUC_0–24h _(ng/mL*h)
Control (blank feed)	22,653 (±16,275)
100 µg·kg^−1^ T-2	29,320 (±13,334)
100 µg·kg^−1^ T-2 + binder	43,961 (±7,982) *
Blank feed + binder	29,343 (±7,681)

#### 3.1.2. Intake of Binder Supplemented Feed Results in Higher Plasma Concentrations of Paromomycin

**Figure 2:**
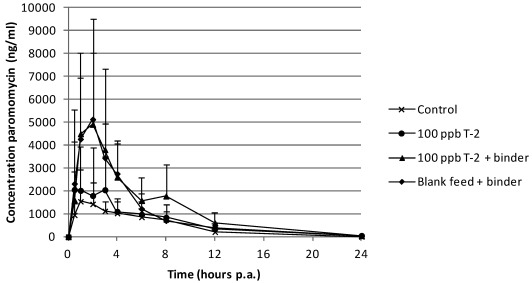
Mean plasma concentrations (+SD) in pigs after single peroral administration of paromomycin at 100 mg kg^−1^ body weight (*n* = 6). Prior to the bolus, pigs received during 7 days, blank feed (control group), feed contaminated with 100 µg T-2 toxin per kg feed, feed contaminated with 100 µg T-2 toxin per kg feed supplemented with mycotoxin binder and blank feed supplemented with mycotoxin binder, respectively.

The plasma concentration-time curve of paromomycin after p.o. administration for the different groups is presented in Figure 2.

Although a trend for higher plasma concentrations after intake of binder supplemented feed was seen, there were no significant differences in plasma concentrations or AUC_0–24h _of paromomycin on the basis of treatment.

### 3.2. Experiment 2

#### 3.2.1. Intake of DON Contaminated Feed Supplemented with Mycotoxin Binder Results in Higher Plasma Concentrations of Doxycycline

**Figure 3:**
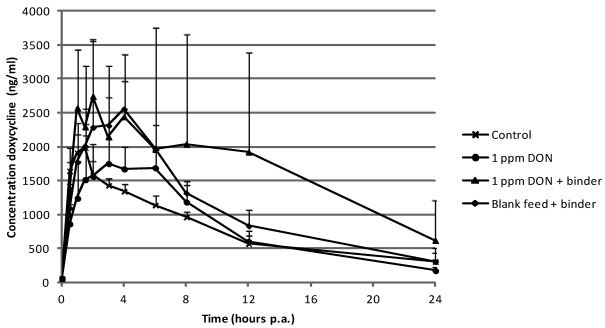
Mean plasma (+SD) concentrations in pigs after single peroral administration of doxycycline at 10 mg kg^−1^ body weight (*n* = 6). Prior to the bolus, pigs received during 13 days, blank feed (control group), feed contaminated with 1 mg DON per kg feed, feed contaminated with 1 mg DON per kg feed supplemented with mycotoxin binder and blank feed supplemented with mycotoxin binder, respectively.

The plasma concentration-time curve for doxycycline after p.o. administration is presented in Figure 3 for the different groups.

The plasma concentration of doxycycline was significantly (*P* = 0.045) higher in the group which received DON contaminated feed supplemented with the mycotoxin binder, but not to the other experimental groups. 

The mean area under the plasma concentration-time curve (AUC_0-24h_) is summarized in Table 2.

**Table 2:** Mean (±SD) area under the plasma concentration-time curve (AUC_0–24h_) for doxycycline in the different groups. Prior to the bolus, pigs received during 13 days, blank feed (control group), feed contaminated with 1 mg DON per kg feed, feed contaminated with 1 mg DON per kg feed supplemented with mycotoxin binder and blank feed supplemented with mycotoxin binder, respectively. Superscript (*) refers to a significant difference compared to the control group (*P* < 0.05).

Group	AUC_0–24h_ (ng/mL*h)
Control (blank feed)	19,011 (±2,805)
1 mg·kg^−1^ DON	20,107 (±3,304)
1 mg·kg^−1^ DON + binder	40,029 (±22,775) *
Blank feed + binder	26,783 (±4,752)

#### 3.2.2. No Statistical Different Plasma Concentrations of Paromomycin after Intake of DON Contaminated Feed

**Figure 4:**
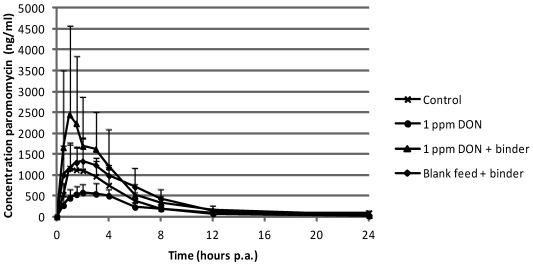
Mean (+SD) plasma concentrations in pigs after single peroral administration of paromomycin at 100 mg·kg^−1^ body weight (*n* = 6). Prior to the bolus, pigs received during 13 days, blank feed (control group), feed contaminated with 1 mg DON per kg feed, feed contaminated with 1 mg DON per kg feed supplemented with mycotoxin binder and blank feed supplemented with mycotoxin binder, respectively.

Plasma concentrations of paromomycin after intake of DON contaminated feed were not significantly different compared to the control group (Figure 4).

## 4. Discussion

The results obtained indicate that there is a need for extensive research into the field of safety testing of mycotoxin detoxifying agents. Indeed, on request of the European Commission, the Panel on Additives and Products or Substances used in Animal Feed derived a proposal for the modification of Annex III of Commission Regulation (EC) No 429/2008 [32]. For the authorization of additives belonging to the functional group of substances for reduction of the contamination of feed by mycotoxins, one of the parameters that needs to be taken into account is the presence and characterization of possible interactions of the binder with, among others, veterinary medicinal products.

In the present study, the effect of mycotoxins and a commonly used mycotoxin binder on the absorption of two frequently used antibiotics in pigs was investigated *in vivo*. The study was conducted in pigs because, among farm animals, pigs react most sensitively to exposure to trichothecene mycotoxins [33]. The antibiotics tested were doxycycline and paromomycin. These veterinary drugs are often used in pigs in ‘mass’ medication and are frequently administered via the oral route, *i.e.*, mixed in the feed or drinking water. The mycotoxins used were the trichothecenes DON and T-2. T-2 toxin is the most potent and toxic trichothecene for which there are no recommended maximum levels in animal feed [24]. The contamination level used in the experiment was based on Monbaliu *et al.* [1] who analyzed 82 feed samples from different European countries for the presence of mycotoxins. As T-2 was found at a concentration ranging from 10–122 µg·kg^−1^ it was decided to produce feed contaminated with 100 µg·kg^−1^. DON on the other hand is one of the most frequent contaminants of maize and small grain cereals with a recommended maximum concentration level of 900 µg·kg^−1^ of DON in pig feed [24]. It was decided to respect this limit and work with a concentration of approximately 1,000 µg·kg^−1^. The mycotoxin adsorbing agent used was an esterified yeast cell wall that claims to bind DON and T-2.

Regarding the plasma concentration of doxycycline, the concentration-time curve of the control group shows a profile as described previously [27]. At 6 h post administration two out of six pigs showed an extra peak in the plasma concentration-time profile. This may be due to the enterohepatic recirculation of doxycycline [34]. Compared to this control group the plasma concentrations were significantly higher in the group which received feed contaminated with 100 µg T-2 kg^−1^ supplemented with the mycotoxin binder. Remarkably, the AUC was almost double compared to the AUC of the control group. Significant higher plasma concentrations of doxycycline were also found when the mycotoxin DON was added together with the mycotoxin binder in the feed. 

In an attempt to clarify the mechanism behind the increased plasma concentrations, a possible interaction of the binder with divalent ions such as Ca^2+^ and Mg^2+^, which normally partially bind doxycycline [35], was presented as potential explanation. With less bound doxycycline present in the intestinal lumen, more doxycycline could then enter the systemic circulation. To check this assumption an extra experiment was performed with oxytetracycline as antibiotic. Oxytetracycline also belongs to the class of tetracyclines like doxycycline and binds even stronger to divalent ions [36]. If our assumption was correct, the effects seen with doxycycline should be even more pronounced with oxytetracycline. However, no statistical difference in plasma oxytetracycline concentration could be demonstrated between the groups receiving either blank feed or blank feed supplemented with binder. In the oxytetracycline experiment however, no T-2 toxin was added to the feed. Therefore, the increased plasma concentrations seen for doxycycline are probably the result of a more complex interaction between mycotoxin, binder and drug. 

For paromomycin, there was no statistical difference in plasma concentrations between the groups. This is possibly due to the great variation in plasma concentration between the individual pigs. Although not significant, there is a tendency that the intake of binder supplemented feed also leads to an increased plasma concentration. 

Recent *in vivo* experiments at our department with broiler chickens also confirm the possible role of binders in the absorption of antibiotics. In chickens, increased plasma concentrations of oxytetracycline were seen after intake of feed supplemented with a mycotoxin detoxifying agent (unpublished data). We also showed that, in chickens, administration of a single bolus of DON combined with a glucomannan binder leads to increased plasma concentrations of DON compared to the control group which received a bolus with only DON (unpublished data). 

The mechanisms for these phenomena still remain unknown but it demonstrates that effects can be binder, species and drug specific. Another possible explanation that we put forward is that the mycotoxin binder affects the intestinal epithelium in which the duration of exposure can play a role, for example by loosening of the tight junctions, stimulating the production of cytokines, influencing the length of villi and depth of crypts, influencing the production of mucus *etc*., which could result in increased passage of antibiotics across the intestinal wall.

In conclusion, our results demonstrate that the studied mycotoxin binder may significantly affect the oral bioavailability of doxycycline. This may be of importance for the animal, the withdrawal time of veterinary drug formulations containing doxycycline, and consequently for public health with respect to tissue residues of the antibiotic. On the other hand, if the mycotoxin binder leads to increased plasma concentrations, this can be compensated by reducing the dosage of the antibiotic. However, since the effects depend on the type of detoxifying agent, type of drug and the animal species, further research is needed to elucidate possible interaction mechanisms.
